# The impact of macroeconomic and structural factors on the unemployment of young women and men

**DOI:** 10.1007/s10644-021-09341-9

**Published:** 2021-07-26

**Authors:** Beata Bal-Domańska

**Affiliations:** Wroclaw University of Economics and Business, Wrocław, Poland

**Keywords:** Youth unemployment, EU countries, Structural change, Panel data, J6, O52, O11, O14

## Abstract

The presented article follows the research mainstream of econometric analyses focused on the assessment of correlations between youth unemployment rate and market and macroeconomic determinants, including economic growth and productivity of the economy, its structure in terms of NACE Rev.2 sections as well as the labor market tools. The research addresses 28 European Union (EU) countries. The analysis period covers the years 2008–2018. The econometric methods dedicated to panel data were used. The research results confirm the importance of the general economic condition as well as the development of knowledge-based economy for the improvement of the youth situation in the labor market. With regard to the economy structure, the development of manufacturing section importance turned out to be a major factor in female youth unemployment rate reduction. The growing importance of the construction sector translated into a decline in the unemployment rate among young men.

## Introduction

Certain groups (including women, young people, people with disabilities, youth with migrant background), which are particularly exposed to the risks of unemployment, long-term unemployment, early school leaving or inactivity, have been experiencing an increasingly difficult situation in many countries over the years. It initiated the debate in scientific literature as well as national and international economic policy, including the European Union (e.g., European Commission 2011).

The successive crises contribute to the deteriorating situation of the youngest participants of the labor market. At the macroeconomic level, failing to integrate the young generation implies a loss of production, productivity and probably also innovation potential (Berlingieri et al. 2014). The situation of young people depends strongly on the processes of globalization, the supply of jobs, qualifications and, obviously, periods of economic prosperity. The ongoing transformation processes and socioeconomic changes result in the need to monitor this problem and refresh the relevant knowledge on a continuous basis.

The purpose of this paper is to assess the situation of young women and men on the domestic labor markets in the context of macroeconomic and structural determinants of national economies. The study analyses 28 EU countries, including the UK. The analysis period covers the years 2008–2018, for which statistical data were available at the time of conducting the research. The study uses the data available in the EUROSTAT database, which ensured their comparability between countries as well as high quality of statistical information. The econometric methods dedicated to panel data were used to characterize relations between the determinants of domestic markets and the situation of young people. It allowed for including the country-specific unobservable effects in the conducted analysis.

When assessing the situation of population on the national labor markets, the macro-solutions influencing a given labor market should be taken into account. The economic and macroeconomic situation expressed through the values of the most important economic parameters shapes the basic conditions for the population functioning on the labor market. In addition, cultural, demographic, political or institutional factors, as well as active and passive labor market policy instruments should be considered along with the extent of skills, job mismatch and spatial mismatches (Bal-Domańska and Sobczak 2018). Additionally, the situation of each person will depend on their individual predispositions, skills and education.

As (Marelli et al. 2013) states, the effect of various macro-factors on youth unemployment is greater than the effect on overall unemployment and the significance between the results for youth and total unemployment rate differs.

Current debates, addressing the employment policy, frequently include gender-specific factors such as human capital accumulation, medical advances, gender-biased technical change, cultural change and antidiscrimination interventions, which imply a rise in the female intensity across the whole economic structure (Ngai and Petrongolo 2017). The presented analysis also relates the problem under consideration to the situation of young women and men.

The presented study is organized as follows. Section 1 provides an introduction into the discussed problem. Section 2 presents the theoretical approach to youth unemployment and the factors determining the position of young women and men on domestic labor markets. Section 3 describes the proposed procedure and research questions, while Sect. 4 addresses YUN (youth unemployment rate), the defined phenomena (factors) used in the analysis, as well as the scope and sources of statistical data. Section 5 outlines the applied statistical approach, while Sect. 6 presents and discusses econometric models being the subject of the essential analysis covering the situation of youth on the domestic labor markets in the EU-28. Section 7 describes the empirical results. The study is concluded with a discussion and an outlook of the current research.

## Factors determining the position of young women and men on domestic labor markets

The factors determining the situation of young women and men on the labor markets can be divided into 6 groups:

- Economic—related to the general situation in the economy and covering such problems as: the level of development and growth rate; productivity,
- Structural and technological change—affecting the supply of jobs (development of service, industrial and agricultural sectors; development of high-tech sectors, dominance of the private sector, dominance of large or small business entities),
- Legal regulations and social policy tools—facilitating the labor market entry for other groups of people (labor market model, flexibility of employment forms, length of working time, minimum wage regulations),
- Education and vocational training systems—related to the adequacy of jobseekers’ qualifications to the existing market needs,
- Cultural—resulting from the division of roles and commitment to work for the family,
- Demographic—related to birth rate, migrations and age structure.

Some of them will affect the situation of women and men in a different way. It also applies to the structural area and labor market tools. The characteristics of individual problem areas are presented below, to the extent needed to carry out further analysis, i.e., focusing on the macroeconomic determinants and the labor market.

### Economic

The factors included in the first group (economic) are predominantly manifested in an increase or decrease in the total number of jobs; however, the changes in the number of jobs have a different effect on the individual groups of people. When analyzing the group of young people, the research results show that the economy development supports job creation and employment, which improves the situation of young people. However, improving the efficiency (labor productivity) can result in the deterioration of youth situation on the labor market, primarily in the short-term perspective. The dependency between youth unemployment and labor productivity differs across countries (economies) (Parisi et al. 2015, 2018). The way in which productivity growth translates into the available jobs depends on the source of productivity growth, whether it is a shift from low-productivity sectors (e.g., agriculture) to high-productivity ones (e.g., industry), or whether it is an increase in productivity recorded in a given economy sector.

Apart from changes in the level of GDP, the level of GDP itself is important as it reflects the level of development and constitutes a kind of approximation for the average wage level. If it is assumed that GDP per capita is a proxy of average labor cost being proportional to those of young and adult groups, than it might be expected to remain positively correlated with youth (and also adult) unemployment. High labor costs may discourage employers from increasing employment and encourage the rationalization oriented activities, e.g., aimed at increasing employee productivity. Thus, employers will not be willing to employ young people without appropriate work experience.

Unemployment rates react strongly to economic cycles, as evidenced by the estimates of so-called Okun coefficient. Okun's law posits an inverse relationship between changes in output (GDP) growth and changes in unemployment. According to Bod’a and Považanová 2020 findings, Okun’s law does tend to vary in strength for genders and for changes in output and unemployment. Okun’s law is found stronger for males than for females, and in the majority of the 21 researched OECD countries in the years 1989–2019, male unemployment is more sensitive to output fluctuations than female unemployment, or there is no significant difference whatsoever. In turn, when assessing the situation of young people and comparing it to that of adults, the researchers concluded that young people are more vulnerable to economic fluctuations with respect to their unemployment. For almost all countries, research confirms a higher youth—rather than total—unemployment rate sensitivity to output gap fluctuations (both positive and negative) (Butkus and Seputiene 2019; Bluedorn et al. 2019; Dunsch 2017) and suggests highest absolute Okun coefficients among youth cohort (Hutengs and Stadtmann 2013b) (Hutengs and Stadtmann 2013a). It is worth emphasizing that “Okun coefficient” shows significant regional differences as well (Kliber 2021), (Bartosik 2020); it may also apply to countries, as a result of which any turbulence in the economy may, to a various extent, be manifested in a deterioration of the situation of young people. As shown (Dunsch 2017) based on the example of Poland and Germany, in Poland the differences between the sensitivity of young people to macroeconomic shocks are greater, whereas in Germany they remain statistically insignificant, and thus, “Polish people are hit harder by macroeconomic shocks in comparison with young German people. This result not only holds for youth, but for all age cohorts”.

### Structural and technological change

Sectoral structure of the economy has a direct impact on the type of available jobs, favoring or hindering access to the desired activities. It is related to the development of structural unemployment, which results from the discrepancy between the supply of labor and the respective demand. The reasons may be attributed to skills mismatch between the jobseekers and the available jobs. This phenomenon may additionally intensify in the situation where jobs usually chosen, e.g., by men dominate the labor market, as in the case of the development of mining. As a result, in a given area, one of the groups (in terms of gender) may present a higher unemployment rate. From the perspective of the economy, the development of modern sectors based on high-technologies and digitization should directly favor the employment of young people who were brought up on the achievements of technology. New technologies as the basis for changes in the sector structure of economies may affect the situation of young people in two ways: (1) positively through opening jobs that favor young people capable of working with new technologies; or (2) negatively by replacing jobs with production automation processes and thus restricting access to jobs for less qualified workers. At this point it is worth recalling the observations presented by (Bluedorn et al. 2019) for the emerging markets and developing economies, which suggest that in the areas being more vulnerable to automation fewer young men—and to a lesser extent, older men—participate or are employed.

Among the EU countries, lower unemployment rate among young people (and lower NEET neither in employment, education or training rate) was recorded in the economies characterized by higher involvement in knowledge and research-based economy, which is characteristic for (and correlated with) better developed economies (Bal-Domańska and Sobczak 2020). Also the regions featuring high share of scientists and engineers in active population (%) constitute a more favorable place for young people to take up professional activity (Bal-Domańska 2018). These results suggest that the development of innovative approaches and modern sectors create strong economic conditions, which are also reflected in an improved situation of young people.

The role of service sector is also crucial in these circumstances (in more advanced countries, the signs of a negative effect of structural change on productivity growth have been observed due to the larger role of services) and the saturation with ICT technologies (van Ark et al. 2004). Young workers can sometimes be perceived as cheap on-the-job learning labor and riskier to substitute ICT-enabled, investments in physical capital goods (or tangible capital), in intangible capital, in total factor productivity. Regardless of the correlation between productivity and employment in the short-term perspective (and the possible temporary employment rate decline), productivity does improve employment rates in the long run; however, social capabilities remain essential to achieve this goal in order to exploit the growth potential and the need for a balanced institutional framework in taking advantage of the existing potential (van Ark et al. 2004).

The observed increase in women’s hours of market work as well as the growth in their relative wages is related, in particular, to the development of the services market. As observed by (Ngai and Petrongolo 2017) for the American market after the Second World War and at the end of the 1960s, there is a clear increase in the share of women in terms of working hours, i.e., *the entire (net) rise in female hours took place in the broad service sector, while the entire (net) fall in male hours took place in goods-producing sectors, including the primary sector, manufacturing, construction, and utilities. This phenomenon can be explained, e.g., by the fact that the production of goods requires the involvement of brawn skills being male domain (men are better endowed of brawn skills than women). Services are relatively less intensive in the use of “brawn” skills, while the introduction of brawn-saving technologies may still retain a comparative advantage in services, related to the more intensive use of communication and interpersonal skills, which cannot be easily automated* (Ngai and Petrongolo 2017).

When analyzing the situation of young women and men on the labor markets, it is worth paying attention to the size and ownership structure of business entities, in particular the role of self-employment and the public sector.

Women are less likely than men to start their own business (Perugini and Signorelli 2010). As results from the information presented by (OECD and The European Commission 2019), *less than one in ten (9.6%) working women were self-employed women in 2018, significantly below the share for men (16.9%). Women have lower self-employment rates across all sectors except Other services. Although gender gap has closed slightly over the past decade, it is due to a decline in the number of self-employed men.* Moreover, self-employed women are less likely to have employees than self-employed men, as well as to be working in personal and household services in 2018 (31.6% men vs. 27.1% women), as Professionals (28.3% vs. 18.2%) or as Service and sales workers (27.0% vs. 11.1%). Apart from women, also youth are under-represented in self-employment (OECD and The European Commission 2019). However, as indicated by (Williams 2004) for USA market it is not clear, whether experience gained in self-employment as a youth is easily transferred to adult self-employment and the long-term “consequences” of self-employment are found to be mixed.

Returning to the review of the selected results presented in (OECD and The European Commission 2019), positive observations were made for the European Union in the area of remuneration. The net median annual income for women and men who worked full time in self-employment was approximately equal in the EU in 2018.

The preferences of women in choosing a job (as an employee and in the service sector) allow adopting that the development of public sector will facilitate the growth of female employment. However, some authors suggest that the access to services mostly supplied by public administrations is usually very difficult at early working ages (Perugini and Signorelli 2010).

To sum up, it is worth highlighting the results presented in the Report (ILO 2017). *Trends from the last decade suggest that prominent growth sectors for young workers include: financial services; trade, hotels and restaurants; transport and storage, information and communications; and health services (including care work and social work activities).*

### Legal regulations and social policy tools

The labor market policy model is also of great importance for the situation of individual groups on the labor markets, including the activities performed by the state and labor market institutions, and carried out at the central and local level using both passive and active instruments aimed at interventions in problem areas which due to the scale or limitations cannot be self-improved within the expected time horizon (Frączek 2015).

The social policy doctrine distinguishes 4 types of labor market policy models: Scandinavian (Nordic), continental (cooperative), Mediterranean (Latin) and liberal (Anglo-Saxon). They differ in terms of the scale of funding tasks within the framework of active labor market policy (ALMP) and the manner of their implementation. Among the above-mentioned groups, the greatest part of GDP is invested in active labor market policy under the Scandinavian model (in 1998–2009 it amounted to 1.19%), whereas in case of the liberal one—the smallest (approx. 0.45%) (Frączek 2015).

The labor market problems also represent the area of activities carried out at the EU level. The vulnerability of the young workers, primarily in times of difficulties and economic crises, contributes to making youth a labor market risk factor. In respect of measures to support job creation and to provide incentives for hiring young people, the EU suggests to reduce non-wage costs and reform employment protection legislation, as well as the excessive rigidities of permanent contracts and provide protection and easier access to those left outside the labor market (Votinius 2014, European Commission 2011). One of the most widespread strategies adopted by the European nations in recent decades is to ameliorate gender-based labor market inequality and to implement legislation aimed at ensuring equal pay and equal employment opportunity. It should be expected that higher funds for the policy focused on, e.g., the situation of women and young people will contribute to the improvement of their position on the labor market.

Along with the growing role of active social policy, a number of studies were developed to assess its impact on the level of unemployment at the micro-level—allowing to evaluate the impact on individual participants in various programs, and at macro-level—to examine the relationship between various aggregate variables, such as unemployment, on the one hand, and ALMP’s on the other, and also incorporating the behavioral effect on non-participants, which is necessary to evaluate the general equilibrium consequences (Calmfors and Skedinger 1995). Nevertheless, with this type of research, the problem of ambiguity regarding the obtained results often arises (Whitehouse 1992), which can be *an argument against putting too much faith in them as the deus ex machina that will solve the European unemployment problem* (Calmfors and Skedinger 1995). However, as suggested by the research results (Whitehouse 1992) covering 13 OECD countries, in the period 1974–1986, and addressing the impact of the solution model referring to legislation and the labor market policy (liberal or collective), the collective model may offer certain positive solutions between relative earnings, on the one hand, and a centralized wage fixation or high levels of government employment on the other. The positive impact on the participation rate of women was also showed for the development of public sector.

In turn, the research conducted among 18 OECD countries, in the period 2005–2013, distinguishing 3 market models: flexible, rigidity and flexicurity, indicates that the majority of the variables (union density, bargaining coverage, replacement rate, duration of unemployment benefits, ALMPs and the wage tax) seem to have the best results on the flexible labor market (Australia, Canada, Japan, New Zealand, Switzerland, the UK and the USA) (Sahnoun and Abdennadher 2019).

When discussing the tools of the labor market policy, it is worth paying attention to flexible forms of employment such as part-time and temporary contracts. It can be shown that they favor an increase in youth employment (Bal-Domańska 2020). However, their impact on the situation of women and men may be different. As indicated in the study by (Perugini and Signorelli 2010) based on the results of the analyses covering the EU regions (NUTS-2 level) in the period 1999–2006, part-time, temporary and self-employment, all of them benefit youth males labor market performance. In the case of women, only part-time employment seems effective in reducing unemployment, whereas regional contexts, where self-employment prevails, remain attributable to higher female unemployment.

The risks resulting from the labor market development for young people with the support of temporary contracts should also be emphasized. The (Bartosik 2020) results show on the example of Poland that unemployment responsiveness to output increased in the years 1996–2018, which witnessed an increased use of temporary contracts. Okun coefficients rise particularly for young and female workers, related to the greater use of temporary contracts among these groups. Spain is another example. It has recorded a particularly strong increase in youth unemployment rate. According to (Berlingieri et al. 2014) in Spain *the transition rate into permanent jobs is very low. This is the result of high employment protection of permanent workers and the liberalization of the use of temporary contracts in the nineties. Working with temporary contracts makes youths very vulnerable to job loss in the case of a recession. This process is reinforced by the lack of representation of temporary and unemployed workers in trade unions. Moreover, the bust of a construction and real estate bubble during the financial crisis reinforced job loss in the construction sector*.

### Knowledge, education and vocational training systems

Higher education is perceived as a prerequisite for providing the most attractive inroad into the upper segments of the occupational structure (individual perspective) and for taking up employment in professions requiring knowledge, thus fostering the development of knowledge-based economy (economic perspective).

Knowledge in the form of education remains the essential element in shaping the professional situation from an individual perspective. Based on the employment rates among young people aged 18–34 in 28 EU countries in 2017, it can be shown that the highest employment rates were recorded for the tertiary education graduates (*EM58*), followed by the secondary education ones (*EM34*), whereas the lowest for young people with less than primary, primary and lower secondary education (levels 0–2) (Bal-Domańska 2020).

Higher education also means a shorter period of transition into the labor market. As indicated by the results available in the Report (ILO 2017), globally, for primary school graduates the transition took, on average, 22.2 months (based on the data from 2012 to 2016), for tertiary graduates it was 8.5 months. The time devoted to searching for a satisfactory job was also shorter in the countries characterized by a higher level of development and in urban areas.

A positive impact of education on professional situation was also presented in the studies by (Barabaschi and Mussida 2016) for four Southern European countries, i.e., Italy, Spain, Portugal and Greece characterized by different labor market institutions, even though similar in their features in the welfare states. They find that education plays a role especially for female selection into employment and for highly educated women means the reduction of occupational segregation (or desegregation). Similar conclusions were formulated by (Ryczkowski and Zinecker 2020) based on the research covering gender unemployment in the Czech and Polish labor market, who indicated that higher education improves primarily the situation of women. They stated that women in Poland with a master level of education had their unemployment risk lowered by 37% (men: 27%) and in the Czech Republic by 44% (men: 33%). In turn, the data presented by (OECD and The European Commission, 2019) confirm a positive impact of education on self-employment (the share of tertiary education graduates self-employed men and women increased, however, was greater among self-employed women than in the case of self-employed men).

Vocational education and training (VET) is also considered highly important in the conducted research, as they reduce youth unemployment by providing them with specific skills, thus smoothing the transition from education to work. VET is seen as a solution against both educational drop-out and youth unemployment having a particularly positive impact on the situation of young women in terms of both employment and wages over the entire career (Korber 2019).

At the same time, it should be highlighted that in the majority of EU countries it is women who present a higher level of education comparing to men, and yet their professional situation is more difficult due to other factors. However, the situation is improving in many respects.

The possibility to continue learning is of greater importance for improving the professional situation among young people. The European regional (NUTS-2) markets with well-developed formal and non-formal adult education and training facilities face reducing the problem of professional and educational inactivity among young people (NEET) (Bal-Domańska 2018). However, it is difficult to confirm this effect regarding the reduction of unemployment rate.

There is still a problem of adjusting youth qualifications to the labor market needs. Especially that the conducted analyses confirm that not only low-qualified young workers face the problem in entering the labor market. The difficulties among young people with integrating into the labor market are the reflection of skills mismatch and spatial mismatch (Berlingieri et al. 2014), (Bal-Domańska and Sobczak 2018). The Pompei and Selezneva 2019 results show that after the outbreak of the crisis and in countries with high education mismatch, there is an additional reduction in unemployment risk for highly educated people.

## The scope of research

The research procedure was initiated with identifying the key problems to be analyzed. Three research questions were formulated:

1. Which of the macroeconomic and structural determinants are significantly related to the situation of young people in the labor market?
2. Does the development of industry sectors, services and the sectors related to new technologies stimulate the reduction of unemployment rate among young people, including women and men, in the cross section of 28 EU countries in 2008–2018?
3. Does self-employment or flexible forms of work contribute to the reduction of unemployment rate among young people, including women and men?

It is not easy to answer the formulated research questions. One of the difficulties is the heterogeneity of political, economic and social determinants prevailing in each state. This situation may translate into problems in identifying common, for all the EU countries, relationships between the economy and youth unemployment.

The statistical analysis included 28 European Union countries (including the UK), i.e., the European Union Member States in the years 2008–2018. The basic analysis period covered 11 years.

The research procedure consisted of the following stages:

1. Defining the phenomena potentially important for the analyzed problem
2. Collecting the statistical data for the analyses and constructing the database
3. Defining models (1–3b) for the unemployment rate of young people, including women and men
4. Econometric analysis covering:
5. preliminary data analysis
6. estimation of models—(1–3a) for the unemployment rate of young people.

## Defining the analyzed phenomena including the scope and sources of data

When describing the situation of young people on the domestic labor markets, the analysis was based on youth unemployment rate indicator YUN (%) covering people aged 15–24 divided into total (*t*), women (*F*) and men (*M*). It represents young people as active job seekers. They are the individuals who, as a rule, have completed their education process and are ready to take up employment, but at the same time do not have extensive professional experience. It is important for them to be able to enter the labor market effectively and gain work experience. Early adult unemployment affects adult live. A long period of looking for a job contributes to poorer motivation in seeking and taking up employment, problems in starting an independent life and a weaker competitive position on the labor market. As suggested by Clark and Lepinteur 2019, past unemployment continues to reduce current well-being, but growing up in a favorable context (high family income, educated and involved parents) significantly reduces unemployment experience. The intergenerational effects of unemployment and educational trajectories are also confirmed by (Lindemann and Gangl 2019). They found that father’s unemployment has a strong negative impact on children’s education decisions.

Youth unemployment is also associated with a number of negative effects for society and economy. There is a fiscal cost due to increased welfare payments. There is a loss of tax revenues, a loss of GDP and other economic benefits like a loss in innovation potential (Berlingieri et al. 2014). In addition, there are social costs such as dissatisfaction, uncertain future employment, crimes, problems related to mental and physical health, and also personal costs connected with interpersonal relations and the sense of rejection or helplessness (Rogozińska-Pawełczyk et al. 2014).

The data on youth were retrieved from the Eurostat database and referred to 28 European Union countries (including the UK, which was the EU Member State in the period covered by the analysis). The years after the economic crisis, i.e., 2008–2018, were selected for the analysis. The choice of the period under study also resulted from the availability of statistical data at the time of the conducting the analysis. There were numerous gaps (e.g., regarding the selected data for the NACE Rev. 2 sections), predominantly in terms of gender. It resulted in the smaller number of the degrees of freedom in the estimated models. Single data gaps were filled with the use of interpolation methods.

**Fig. 1:**
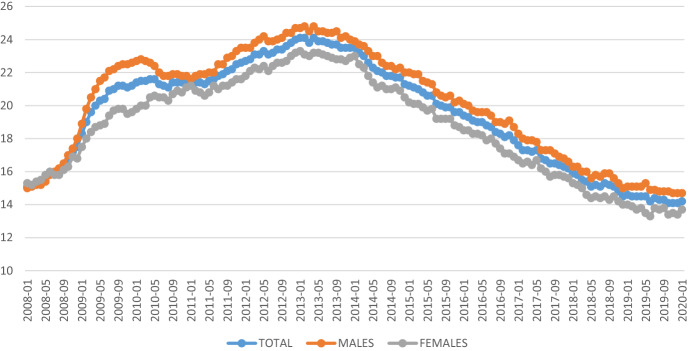
Youth unemployment rate (aged 15–24) in the EU-28 in the period 2008–2020 (monthly data). *Source* author’s compilation based on Eurostat data in MC EXCEL

The analyzed years cover the period the beginning of which is associated with a drastic deterioration of both average and maximum levels of youth unemployment rates: following the financial and debt crisis (in 2009, YUN values at the value of 20.2% for the EU-28 ranged from 10.2% up to 37.7%). The subsequent years represent the period of overcoming the crisis. A clear improvement in youth employment rates was recorded after 2013 (Fig. 1) and lasted until 2020, when the COVID-19 pandemic resulted in a significant increase in youth unemployment rate.

An improvement in the unemployment rate came with a certain delay in relation to the economy. In 2010, only two countries (Croatia and Greece) recorded a decline in GDP. In turn, an increase in the unemployment rate of young people was observed in 18 EU countries.

When assessing two borderline years of the study (2008 and 2018), attention should be paid to the diversified degree of YUN reduction in the individual EU countries. In 10 of them, YUN value increased in 2018 against 2008. It means that despite intensive efforts to improve the condition of economies and the labor market after the 2008 economic crisis, not all countries managed to return to the situation preceding the crisis. The largest growth was recorded in the following order: Cyprus, Ireland, Portugal, Greece, Italy, Spain, Denmark. Ultimately, in 2018, in the EU-28 YUN was at the level of 12.5%, ranging from 6.2% up to 39.9%. In 3 countries (Greece, Spain, Italy), youth unemployment rate exceeded 30%.

High unemployment levels were characteristic only for a part of the EU countries. In some countries, the situation of unemployed youth was good throughout the analyzed period. Germany (YUN 5.8–11.2%) and Austria (YUN 8.5–11.2%) were among these countries.

**Fig. 2:**
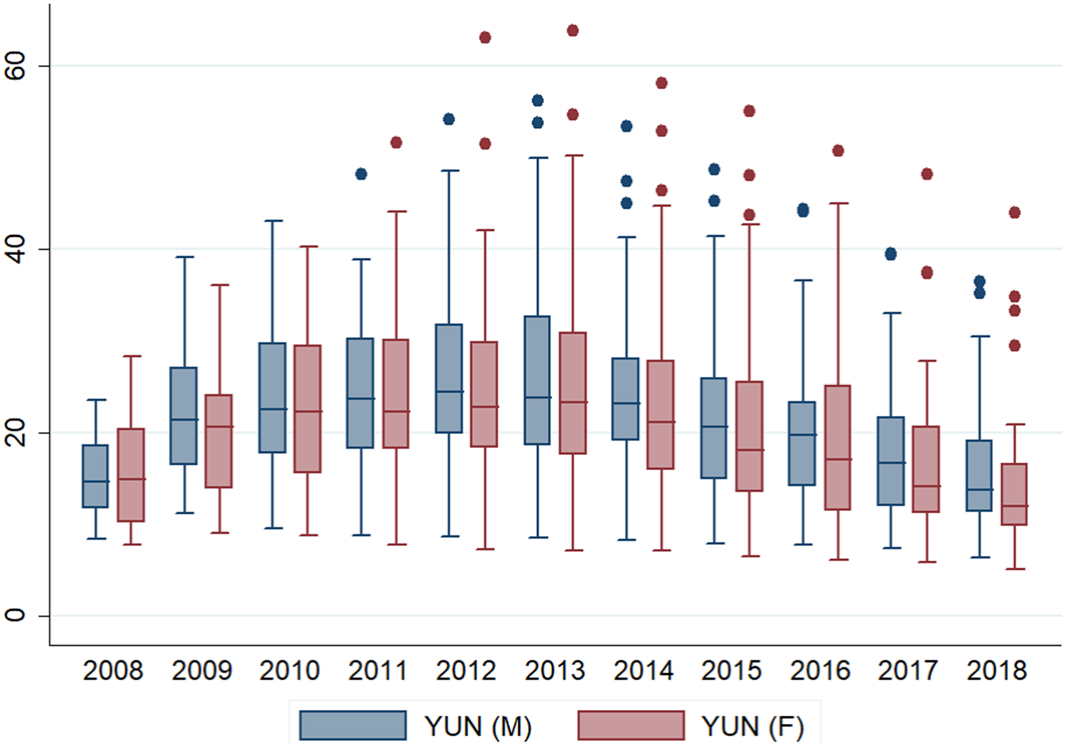
Diversification of youth unemployment rate of men and women in the EU-28 in the period 2008–2018. *Explanation:* the line in the middle of the box presents median value; the upper line of the box is the 75th percentile, the bottom line of the box presents the 25th percentile; a dot is the outside value. Whiskers ended by lower-higher adjacent value. *Source* author’s compilation based on Eurostat data in STATA 10

Taking into account the differences in the unemployment rate of young women and men, the situation among countries is more diversified (particularly after 2009) (Fig. 2). Referring to the value of the coefficient of variation (established as the ratio of the standard deviation and the arithmetic mean), in 2008 the variation in total unemployment rate of young people was 32%, indicating clear differences between countries, at the end of the analysis period, in 2018, the level of variation increased to 53%. Regarding the unemployment rate of young women, the disparities between countries were even greater, the coefficient of variation amounted to 39% in 2008 and 60% in 2018, while for men it was 28% and 49%, respectively. The observed increase in disproportions resulted largely from the situation in a few countries that did not regain stable situation after the 2008 crisis. In Fig. 2, they are shown as dots. These countries include Greece, Italy, Spain and, in terms of women, Croatia.

As a rule, in the studied period at the level of EU-28, the unemployment rate of young men was higher by about 10% than the unemployment rate of young women. Much greater differences were observed in the cross section of countries. In the last year of the study, the difference was as follows: Croatia—50%, Greece—21%, Slovenia—16%, Italy—14%, Slovakia—13%, Czechia—13%. Higher rates of men than women were also recorded in: Hungary (9%), Portugal (6%) and Poland (5%).

When defining the factors included in the model structure, the majority of problems identified in the section *Factors determining the position of young women and men on domestic labor markets* were addressed. The source of data was not found for all of them. Part of the identified information was finally deleted from the structure due to the correlation with other measures, or the absence of (statistical) significance shown at the stage of the preliminary analysis (e.g., the percentage of people working from home, fertility rate). Ultimately, among the identified phenomena considered potentially significant for the situation of young women and men, the variables were indicated in 5 thematic groups:

- Economy—GDP per capita, productivity of the economy,
- Structure of the economy covering 10 NACE Rev. 2 sections (*C*, *F*, *G*, *H*, *I*, *J*, *N*, *O*, *P* and *Q*) and also the information about self-employment (SELF), human resources in science and technology (HRST),
- Labor market and its tools—unemployment rate of adults, ALMP expenditure as percentage of GDP; temporary and part-time contracts as % of total employment,
- Educational capital—share of higher education graduates, early leavers from education and training (%).

The list of variables with the symbol of the source table (EUROSTAT) is presented in Annex.

## Estimation methods

Data and panel techniques were used to estimate models, which allowed including unobserved individual effects (*a*_*i*_) for each country. The general linear model is:

$$Y\left( {{\text{YUN}}} \right)_{{{\text{it}}}} = a_{i} + \, \sum {\beta_{i} x_{{{\text{it}}}} } + \varepsilon_{{{\text{it}}}}$$

where *Y*—dependent variable, *X*—independent variable, *β*_*i*_—coefficients, *a*_*i*_—unobserved country-specific effects, *ε*—random factor.

The models were estimated using LSDV (*least square dummy variable*) estimator (FE model), which enables the exploration of changes over time, taking place in many territorial units (countries), or random effects model (RE). It has been tested whether there is a contextual effect, or whether the between and within effects are different. If high values of correlations between the errors and the regressors were recorded, the fixed effect model was suggested. In the remaining cases—as suggested by the Hausman test (Hausman 1978)—the RE model (random effects) was used. The description of methods is presented in Wooldridge  2010, Greene 2000, Bell et al. 2019.

The regression is based on unbalanced panel data. Models were estimated as the multi-variable regression (see the next section: *Macroeconomic models of youth situation on the labor market*).

Coefficients of determination *R*^2^ (overall, within and between) were used to assess models’ quality and define the role of variance within and between. The between *R*^2^ indicates how much variance between separate panel units the model explains. The within *R*^2^ is a measure of the variance within the panel units (countries). The overall *R*^2^ is the proportion of the variance in the dependent variable which is predictable from the independent variable(s) and is a weighted average of the between and within *R*^2^.

Additionally, Akaike Information Criterion (AIC) and Bayesian Information Criterion (BIC) were used to assess the quality of models. Following the adopted convention, the best model is the one for which the criterion of information value remains the lowest.

Moreover, the conclusions were formulated based on the classical statistical significance test of model coefficients with the null hypothesis stating that the value of *β*_*i*_ is statistically significant at the level of $0.1.$

The validity of introducing $\alpha_{i }$ individual effects into the model structure was assessed using F test with null hypothesis, i.e., all coefficients in the model equal zero.

A fairly common problem in the estimation of panel models is the presence of heteroscedasticity (non-constant variance). Therefore, robust standard errors (Huber/White/sandwich estimator) were used in the interpretation.

All calculations were performed using STATA 10 program.

## Macroeconomic models of the youth situation on the labor market

For the selected phenomena (YUN), econometric models which take into account the structure of the economy, labor market policy tools and the youth education level were proposed. Most of the variables presented values for people aged 15–24, also broken down by gender.

The youth unemployment models were defined as follows:

1 $${\text{YUN}}_{{{\text{it}}}} = a_{i} + \beta_{1} {\text{GDP}}_{{{\text{it}}}} + \beta_{2} {\text{ELne}}_{{{\text{it}}}} + \beta_{3} {\text{PART}}_{{{\text{it}}}} + \beta_{4} {\text{TEMP}}_{{{\text{it}}}} + \varepsilon_{{{\text{it}}}}$$

2 $${\text{YUN}}_{{{\text{it}}}} = a_{i} + \beta_{1} {\text{AUN}}_{{{\text{it}}}} + \beta_{2} {\text{ALMP}}_{{{\text{it}}}} + \varepsilon_{{{\text{it}}}}$$

In addition, the youth unemployment rate models broken down by gender were developed:

3 $$\begin{aligned} {\text{YUN}}_{{{\text{it}}}} = & \, a_{i} + \beta_{1} {\text{PROD}}_{{{\text{it}}}} + + \beta_{2} {\text{HRST}}_{{{\text{it}}}} + \beta_{3} {\text{SELF}}_{i} + \beta_{4} {\text{PART}}_{{{\text{it}}}} + \beta_{5} {\text{TEMP}}_{{{\text{it}}}} + \, + \beta_{6} {\text{TER}}_{{{\text{it}}}} + \beta_{7} C_{{{\text{it}}}} + \beta_{8} F_{{{\text{it}}}} \\ + & \beta_{9} H_{{{\text{it}}}} + \beta_{10} J_{{{\text{it}}}} + \beta_{11} I_{{{\text{it}}}} + \beta_{12} G_{{{\text{it}}}} + \beta_{13} N_{{{\text{it}}}} + \, \beta_{14} O_{{{\text{it}}}} + \beta_{15} P_{{{\text{it}}}} + \beta_{16} Q_{{{\text{it}}}} + \varepsilon_{{{\text{it}}}} \\ \end{aligned}$$

3a $${\text{YUN}}_{{{\text{it}}}} = {\text{stepwise regression}}$$

where YUN*—*youth unemployment rate aged 15–24 (%), AUN*—*unemployment rate aged 25 year and over (%), GDP*—*gross domestic product at current market prices; purchasing power standard (PPS) per inhabitant, PROD*—*gross domestic product at market prices current prices; million purchasing power standards per employed person (resident population concept—LFS), ALMP*—*labor market policy expenditure by type of action; percentage of GDP (categories 2–7) (%), HRST—persons employed in science and technology as percentage of active population, PART—part-time employment as percentage of the total employment for young people; aged 15–24 (%), TEMP—temporary contracts—annual data aged 15–24; percentage of total employment, ELne*—*early leavers from education and training—persons not employed persons aged 18–24 (%), TER*—*young people completed tertiary education level (levels 5–8) aged 15–24 (%), *C*, *F*, *G*, *H*, *I*, *J*, *N*, *O*, *P* and *Q*—share of employed in a given NACE Rev. 2 section (NACE) aged 15–24 in total employment in this age group.

The structure of the models included the value of gross domestic product per capita (GDP), which is designed to reflect the economic situation. An improvement in their value is related to an increase in the demand for jobs and thus supports the employment of young people.

The situation of young people is usually strongly related to the overall situation on the labor market. As the observations for the years 1999–2006 show, youth unemployment rate in the EU regions was, on average, twice as high as the adult unemployment rate (Perugini and Signorelli 2010). During the period under study, in some countries, the ratio of youth unemployment (aged 15–24) compared to that of adults (aged 25 and over) exceeded the value of 3. Sweden, Romania and Luxembourg were listed among the countries presenting the highest levels of this ratio. The opposite side covered countries such as Germany, Slovenia and Latvia, where this ratio often did not exceed 2. To determine the ratio of unemployment between adults and young people, the specification (2) referred to adult unemployment rates for those aged 25 or over (AUN). At this point, the information was obtained how the change in adult unemployment rate translates into youth unemployment rate.

Within the framework of the economic policies aimed at the labor market development, the model in specification (2) included ALMP (active local market policy) variable expressed as the percentage of GDP. ALMP variable takes into account the scale of expenditure on: training, employment incentives, supported employment and rehabilitation, direct job creation, start-up incentives. It was adopted that the increase in outlays on professional activation should be manifested in the declining unemployment also among young people.

In addition, models (1) and (3–3a) included the variables presenting flexible forms of part-time work (PART) and temporary contracts (TEM^*9*^). It was assumed that their greater market share means deterioration of employment forms; however, at the same time it should contribute to the reduction of unemployment rate.

The final specification referring to youth unemployment rate (YUN) (models (3–3a) was developed based on the information on productivity (PROD), human resources in science and technology (HRST) and the economy structure considering the selected sectors, the scale of self-employment (SELF) and flexible forms of employment, as well as considering the education level of young people (TER). These models were constructed for the entire population of the unemployed and also broken down by gender. Additionally, the version (3a) of the model was developed based on a stepwise regression procedure. Student’s t-test statistics for individual coefficients and values of the Akaike Information Criterion (AIC) and Bayesian Information Criterion (BIC) were used as the model selection criteria.

Productivity *(*PROD*)* was defined as gross domestic product at market prices (current prices) in million purchasing power standards per total employment (resident population concept—LFS). It was adopted that higher productivity over time in individual countries will contribute to the economic situation improvement, and thus to higher employment, also among young people (although—as mentioned above—in the short term it may result in the deterioration of youth situation on the labor market. Deterioration of the situation is more probable in the scale of a single company or sector; ultimately, the work assumes a positive correlation between productivity and the situation of young people at the macro-level). Moreover, the increase in productivity also reflects the economy transformation process toward new technologies and frequently also the jobs offering good employment conditions, including the financial ones. In the long run, this factor may encourage young people to make effort and take up work, primarily in the groups which remain inactive as a result of no access to jobs enabling them to fulfill their professional and financial ambitions. The economy transformation towards high technologies and the involvement of human capital with specialized knowledge is represented in the model by the variable of human resources in science and technology (HRST), indicating the level of human capital development in individual countries. It will be related to the economy productivity. High-productivity economies tend to be successful at using technologies implemented by people with expertise knowledge. All individuals meeting the criteria of human resources in science and technology can participate in R&D work, which is manifested in economic development based on knowledge and technology.

Among the variables illustrating the structure, the percentage of the employed in 10 sections of the economy was taken into account in accordance with the statistical classification of economic activities in the European Community NACE Rev. 2 including the share of the employed aged 15–24 in total employment in this age group in the following sections: (*C*) manufacture, (*F*) construction, (*H*) transportation and storage, (*I*) accommodation and food service activities, (*J*) information and communication, (*G*) wholesale and retail trade; repair of motor vehicles and motorcycles, (*N*) administrative and support service activities, (*O*) public administration and defense; compulsory social security, (*P*) education, (*Q*) human health and social work activities.

Unfortunately, the selection of sectors was also influenced by the availability of data for young people. In many cases, the breakdown by gender was connected with numerous data gaps, which resulted in a smaller number of available observations and, hence, the degrees of freedom. Another criterion was the potential significance for the employment of youth (e.g., construction or wholesale and retail trade, or the public sector in the case of women). It was assumed that changes in the sectors sensitive to the situation of youth will react strongly and increasing employment in these sectors should result in lower unemployment among young people. All of the 10 variables for NACE Rev. 2 sections are included in the total version or broken down into male (*M*) and female (*F*).

The situation of young men and women was also characterized in the models by the variables describing flexible forms of employment and SELF variable. SELF variable stands for the percentage of self-employed aged 15–24 (total/female/male). It was assumed that the increase in self-employment should reduce the unemployment rate of young people (similar to flexible forms of employment that characterize young people aged 15–24 employed based on part-time contracts (PART) and temporary ones (TEMP)).

## The results of econometric analysis

Among the factors determining youth unemployment rate, it is worth paying attention to the ones adopted by women and men to a different extent. Their brief characteristics are given below, based on the data for the EU-28 in 2018.

Youth self-employment was most popular in Italy, Romania and Slovakia (approx. 11%), whereas the least popular in: Ireland, Germany, Austria and Denmark. In the case of youth self-employment, the predominance of men over women in using this form of employment is observed (M: 5.2% and F: 2.9%). Particularly large differences in the level of SELF indicator between women and men were noticeable in Romania (M: 13.9% and F: 6.3%), as well as Slovakia, the UK, Greece and Czechia.^1^ It is also worth highlighting that although the average percentage values of self-employed youth in the EU-28 did not change significantly in the initial and final period of the study (Table 1), the situation was more dynamic across countries and 5 countries recorded over 40% increase in the percentage of self-employed young people (Slovenia, France, Slovakia, Czechia and Latvia).

**Table 1:** Values for the EU-28 and coefficient of variation (V) for the variables included in econometric models by gender n 2008 and 2018

	Total			Female			Male		
	2008	2018		2008	2018		2008	2018	
			*V* (%)			*V* (%)			*V* (%)
*YUN*_it_	15.8	15.1	53	15.8	14.5	60	15.9	15.7	49
*PART*_it_	26.0	32.2	60	34.2	40.5	55	19.3	25.2	66
*TEMP*_it_	37.5	40.9	56	38.6	42.0	59	36.5	39.9	57
*SELF*_it_	4.1	4.1	64	3.0*	2.9*	57*	4.9*	5.2*	61*
C_it_	16.3	14.4	52	10.2	8.2	54	21.5	19.7	50
*F*_it_	9.9	6.4	33	1.5*	1.2*	39*	16.8	10.8	31
*G*_it_	21.5	20.4	23	24.7	22.3	21	18.9	18.8	29
*H*_it_	3.6	3.9	30	2.1	2.2	39*	4.8	5.3	28
*I*_it_	9.1	11.9	40	11.4	13.8	34	7.2	10.3	55
*J*_it_	2.7	2.6	33	2.2*	1.8*	27*	3.1	3.3	36
*N*_it_	4.0	4.2	38	4.0	3.9*	30*	3.9	4.4	42
*O*_it_	4.0	3.6	58	3.8*	3.7*	52*	4.1	3.5	67
*P*_it_	3.3	4.4	41	5.1	6.7	34	1.7	2.5	50
*Q*_it_	7.3	8.9	54	13.3	15.5	47	2.4*	3.3*	47
*ELne*_it_	6.7	5.6	45	7.2	5.5	56	6.2	5.8	42
*TER*_it_	7.3	9.8	51	8.8	11.5	49	5.8	8.1	57

*V* Coefficient of variation based on standard deviation and mean value. *The value determined at large amount of missing information

*Source* Author’s compilation based on EUROSTAT data

In turn, part-time and temporary contacts were more popular among women. The predominance of women over men in the case of part-time contracts was significant (*M*: 16.9% and *F*: 31.3%), as opposed to temporary contacts, which presented a similar level at the EU level (*M*: 31.2% and *F*: 33.2%). Among some EU countries, the level of part-time contracts popularity was relatively high and additionally characterized by large differences between women and men (Netherlands, Denmark and Sweden).

**Fig. 3:**
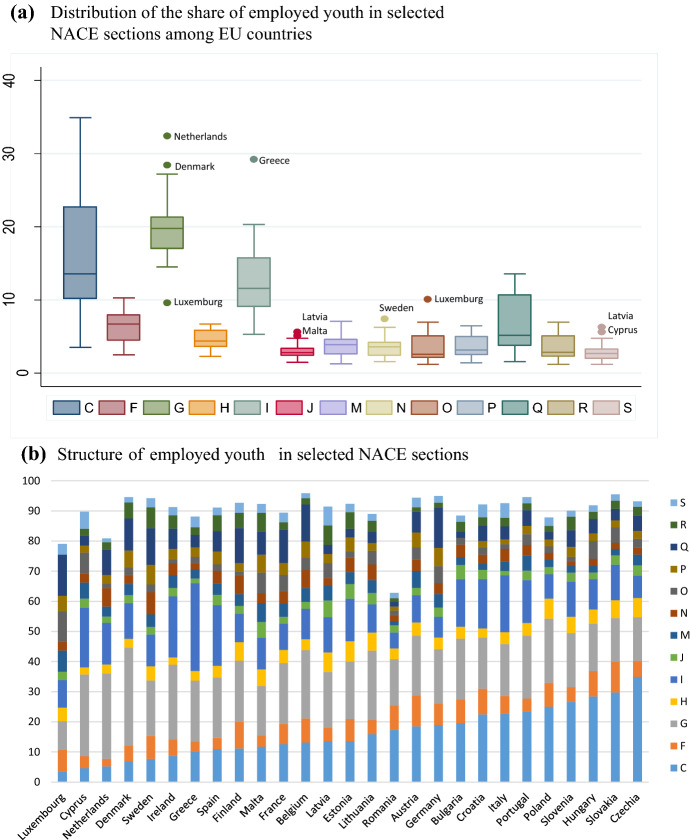
Share of the employed (aged 15–24) in particular NACE Rev. 2 sections for the EU-28 in 2018.Due to the lack of data for 2018, the data from previous years were used in 7 cases. For Bulgaria no data for *P* section, and for Luxemburg—*R* section. *Source* author’s work based on EUROSTAT data in Excel and STATA 10 programs

The economy structure has a direct impact on the availability of jobs meeting the preferences of women or men. When analyzing data for the EU-28 in 2018, 5 NACE sections can be identified, where young people find employment most frequently (Fig. 3a). They are as follows: (*G*) wholesale and retail trade; repair of motor vehicles and motorcycles, (*C*) manufacturing, (*I*) accommodation and food service activities, (*F*) construction, (*Q*) human health and social work activities. Women and men choose the activities representing different sections. This translates into extensive differences in the number of employed women and men. Two of the listed sections are most popular among men (*F* and *C*), whereas women work more often in the remaining sections: *G*, *I*, *Q* (Table 1).

It is also worth paying attention to the differences in the youth employment structure between countries. It primarily refers to section *C* (manufacturing), for which the range between the minimum and maximum share of the employed is the biggest (Fig. 3a). The share of youth employment differs in this case from only 3.5% (Luxemburg) and 4.6% (Cyprus) to over 25% (Poland, Slovenia, Hungary, Slovakia, Czechia). We observe a kind of an interchangeability between youth employment in C (more popular in Eastern Europe) section and *G* and *I* (Western Europe).

Huge disproportions are also observed for section I (accommodation and food services); in this case the share of youth ranges from 5.3% in Romania to almost 30% in Greece (which is an outstanding observation marked dot). Also *Q* section (human health and social work activities) varied between countries significantly recording the lowest value for Central and Eastern European countries (Romania, Bulgaria, Poland, Estonia, Latvia), and the highest values for Western European countries (Netherland, Denmark, France, Finland, Sweden, Belgium, Germany, Luxemburg).

Comparing the importance of individual sections of the economy in creating jobs for young people in the final and initial years, it should be noted that after the disruptions on the labor market caused by the 2008 economic crisis, the structure of youth employment in the economy has changed. The role of manufacturing (*C*),^2^ construction (*F*)^3^ and also slightly wholesale and retail trade (*G*) sectors has declined (at the EU level). At the same time, the percentage of people employed in such sections as accommodation and food service activities (*I*), education (*P*) and public administration and defense (*O*) went up. Unfortunately, in the case of young people working in the sector and due to the current situation resulting from the COVID-19 epidemic, the risk of losing employment was particularly high.

The econometric analysis results confirmed some of the assumptions made regarding the relations between youth unemployment rate and the analyzed socioeconomic factors, as well as unexpected findings.

In general terms, the obtained estimations of econometric models (1–3a) were characterized by the following properties:

- Test *F*, at each statistical significance level, allowed for rejecting *H*_0_ about the insignificance of country-specific coefficients *α*_*i*_ in the model, showing that they are different than zero,
- High values of intraclass correlations were obtained in all models. It means that over 90% of the variance results from the differences across panels (countries),
- In models (1) and (3), high values of correlations between the errors and the regressors were recorded (over − 0.8). It confirms the grounds for using the fixed effects FE approach (instead of a random effects model).
- Model (2) was estimated based on RE approach.

Based on the estimation of models in accordance with the specification (1), the statistical significance of the economic development (GDP) was confirmed in explaining the changes in youth unemployment rate (YUN), with this factor being significant for explaining the changes occurring within each country (Table 2). This, however, is a rather small effect. Thus, promoting economic growth alone does not stimulate strongly the labor demand for young people. The positive, even though small, impact of GDP on the situation of young people is consistent with the findings collected by other researchers (Eurofound 2012).

**Table 2:** The results of multivariable estimation of models (1–3a) for the EU-28 in the period 2008–2018

	(1) Fixed effect	(2) Random effect	(3) Fixed effect	(3a) Fixed effect
*PROD*_it_			− **0.174** [0.045]	− **0.178** [0.041]
*GDP*_it_	[0.008]			
*UNA*_it_		**2.230** [0.000]		
*ALMP*_it_		− **2.314** [0.077]		
*HRST*_it_			− **0.893** [0.002]	− **0.910** [0.001]
*PART*_it_	**0.829** [0.000]		**0.614** [0.001]	**0.603** [0.001]
*TEMP*_it_	0.117 [0.199]		− 0.056 [0.689]	
*SELF*_it_			− **2.007** [0.000]	− **2.019** [0.000]
*ELne*_it_	**1.706** [0.003]			
*TER*_it_			0.079 [0.600]	0.095 [0.501]
C_it_			− **0.510** [0.047]	− **0.518** [0.007]
*F*_it_			− **0.920** [0.026]	− **0.825** [0.028]
*G*_it_			− 0.010 [0.977]	
*H*_it_			− 0.613 [0.216]	− 0.629 [0.231]
*I*_it_			− 0.065 [0.844]	
*J*_it_			− **1.813** [0.004]	− **1.856** [0.001]
*N*_it_			0.761 [0.192]	0.700 [0.178]
*O*_it_			0.572 [0.481]	0.587 [0.439]
*P*_it_			0.256 [0.667]	0.250 [0.650]
*Q*_it_			0.606 [0.142]	0.638 [0.193]
*R*^2^ within	0.499	0.895	0.623	0.622
*R*^2^ between	0.013	0.787	0.017	0.010
*R*^2^ overall	0.034	0.820	0.0001	0.0002
AIC *BIC*	1740.9 1755.8	x x	1351.9 1408.5	1346.7 1392.7
Number of observ.(*i*)	308 (28)	308 (28)	254 (26)	254 (26)

Bold values are statistically significant at the level of 0.1

[] p-value based on robust standard errors

*Source* author’s compilation in STATA 10

The effect of youth unemployment rate reduction is most frequently attributed to flexible forms of employment, also defined as the opportunity to bridge the so-called experience gap (Perugini and Signorelli 2010). Greater interest in part-time and temporary contracts may take place in the periods of crisis, which was observed in the EU countries in the period under study (2009–2018) (Table 1). In most countries, an increased popularity of part-time contracts was recorded among young people, predominantly in the countries where their significance was low at the beginning of the discussed period (2008) (e.g., Slovakia, Croatia). Their role was slightly lower in Romania and Swede alone. It should be noted that Sweden is one of the EU countries presenting one of the highest shares of part-time contracts among youth (in 2018 PART variable reached 44.3%).

In the presented analysis, formulating correlations between PART and TEMP and the unemployment rate turned out to be more problematic. A positive sign was obtained for part-time contracts, whereas temporary contracts did not show any statistically significant influence. Due to the fact that part-time contracts (PART) are mainly valid in explaining differences within groups, it implies that in the EU countries, in the years 2008–2018, the growing interest in part-time contracts was accompanied by an increase in youth unemployment rate, and thus, a certain “deterioration” of working conditions was observed.

The factors included in the specification (1) allowed explaining, to a small extent, the overall youth unemployment rate (YUN) variance. The highest level of explanation was obtained for the variance within the panel units (countries) (within *R*^2^ = 50%). The factors included in model (1) are responsible, to the greatest extent, for the development of the situation in the EU countries.

Model (1) confirms the positive impact of reducing the share of not employed early leavers in the population of young people on the reduction of the unemployment rate in this cohort. It is worth noting that in 2018, compared to 2008, the ELne index decreased (improved) in most 28 EU countries. However, eight countries failed to reduce the number of people in the ELne group. For 4 of these countries (Italy, Croatia, Romania and Finland), the situation seems extremely difficult, as these countries are characterized by high unemployment rate among young people and simultaneously the highest percentage of not employed early leavers in the EU.

The statistically significant influence of ALMP variable was observed in the specification (2). A negative coefficient obtained for ALMP variable stands for the positive impact of ALMP expenditure on youth unemployment scale reduction. At the same time, the unemployment rate of adults AUN (aged over 25) was included in this specification. It has been shown that the situation of youth is correlated with the general situation on the labor market and is usually 2.235 times more difficult than the situation of adults. The determinants of the labor market, presented in terms of adult unemployment rate, allow explaining the changes in the situation of young people on a given labor market (within differences), but also the differences between the particular markets (between differences). The coefficient of determination indicates that the situation of youth can be explained at the level of 90% by the current situation on a given labor market, while 79% is explained by the differences between the domestic labor markets.

The specifications (3) and (3a) were focused on assessing relations between the situation of youth (YUN) and the productivity and structure of the economy. The estimation results (Table 3) allowed formulating the following conclusions:

- Improving the productivity of the economy facilitates the reduction of youth unemployment rate,
- The development of human resources in science and technology (HRST) facilitates the reduction of youth unemployment rate to a greater extent than productivity,
- Self-employment and the development of youth employment in construction (*F*), information and communication (*J)* as well as manufacturing (*C*) sections improved the situation of youth on the labor market,
- The increase in part-time contracts changes across time and between countries by one unit was associated with higher youth unemployment rate,
- Temporary contracts show a statistically insignificant relation,
- The share of very young people who completed tertiary education level does not present any statistically significant relation.

**Table 3:** The results of multivariable fixed effect estimation of models (3 and 3a) by gender for the EU-28 in the period 2008–2018

	(3) YUN_it_	(3a) YUN_it_	(3b) YUN_it_	(3) YUN_it_	(3b) YUN_it_
	Female			Male	
PROD_it_	− **0.355** [0.075]	− **0.370** [0.052]	− **0.627** [0.000]	− 0.127 [0.311]	− **0.327** [0.000]
HRST_it_	− 0.716 [0.128]	− **0.692** [0.095]		− **0.813** [0.096]	
PART_it_ (*F*/*M*)	**0.741** [0.004]	**0.736** [0.001]	**0.920** [0.002]	0.095 [0.719]	0.176 [0.585]
TEMP_it_ (*F*/*M*)	− 0.038 [0.846]		0.029 [0.882]	− 0.185 [0.377]	− 0.135 [0.583]
SELF_it_ (*F*/*M*)	− 1.112 [0.134]	− 1.121 [0.131]	− **1.346** [0.069]	− 1.471 [0.113]	− **1.889** [0.028]
TER_it_ (*F*/*M*)	0.015 [0.928]		− 0.084 [0.659]	0.302 [0.103]	0.126 [0.304]
*C*_it_ (*F*/*M*)	− **1.581** [0.002]	− **1.606** [0.004]	− **1.500** [0.008]	− 0.438 [0.295]	− 0.250 [0.368]
*F*_it_ (*F*/*M*)	x	x	x	− **0.964** [0.028]	− **1.047** [0.004]
*G*_it_ (*F*/*M*)	− 0.213 [0.579]	− 0.221 [0.558]	− 0.120 [0.747]	0.394 [0.508]	0.590 [0.363]
*H*_it_ (*F*/*M*)	− **0.183** [**0.079**]	− **1.142** [0.069]	− 0.586 [0.375]	− 0.744 [0.225]	− 0.688 [0.347]
*I*_it_ (*F*/*M*)	− 0.169 [0.739]	− 0.221 [0.617]	− 0.429 [0.417]	0.188 [0.798]	
*J*_it_ (*F*/*M*)	− 1.740 [0.165]	− 1.718 [0.185]	− 1.646 [0.171]	− 0.831 [0.194]	− 0.840 [0.259]
*N*_it_* (F/M)*	− 0.279 [0.710]	− 0.253 [0.718]	− 0.087 [0.907]	0.102 [0.845]	− 0.042 [0.938]
*O*_it_* (F/M)*	− 1.327 [0.162]	− 1.319 [0.166]	− 1.048 [0.283]	0.427 [0.682]	0.759 [0.381]
*P*_it_* (F/M)*	0.591 [0.470]	0.611 [0.422]	0.544 [0.533]	− 0.105 [0.889]	− 0.228 [0.797]
*Q*_it_* (F/M)*	0.308 [0.338]	0.311 [0.291]	0.316 [0.324]	0.234 [0.750]	− 0.146 [0.842]
AIC	613.6	613.8	624.5	679.5	698.5
BIC	647.3	647.5	658.3	723.7	739.7
*R*^2^ within	0.643	0.624	0.610	0.627	0.567
*R*^2^ between	0.025	0.026	0.000	0.0001	0.106
*R*^2^ overall	0.026	0.026	0.0003	0.019	0.035
Number of observations (*i*)	123 (15)	123 (15)	123 (15)	140 (16)	140 (16)

Bold values are statistically significant at the level of 0.1

[] robust standard errors

*Source* author’s compilation in STATA 10

The discussed changes in YUN analyzed in the specifications (3–3a) resulted, to a great extent, from the variance within the panel units (countries) (within *R*^2^ about 62%), along with the simultaneous low level of the variance between panel units (countries) (between *R*^2^ from 1 to 2%), which for the collected findings highlights the great role of activities carried out within individual countries.

The re-formulation of models (3) and (3a) for women and men (Table 3) allowed obtaining additional information. Compared to the models for the entire population (Table 2), certain problems with introducing the HRST variable were recorded and thus in some specifications it resulted in no statistical significance of the PROD variable. Finally, Table 3 presents the results for the specification (3), as well as the model for women after performing step regression (3a) (for men, the specification (3) turned out to be the best in the light of the AIC and BIC criteria). Additionally, the results of step regression for the specification without the HRST variable (3b) are presented.

A certain limitation of the results presented in Table 3 takes the form of the varying number of observations underlying each model. Unfortunately, the availability of statistical data broken down by gender for each NACE section turned out to be limited, which resulted in a different number of the degrees of freedom in individual models and a limited number of the included countries (from 15 to 16). Nevertheless, certain generalizations can be formulated based on the research findings. In the case of the variable reflecting the share of female employment in section *F* construction, less than 50% of the data were available; therefore, this variable was ignored in the specification (3) for women; thus, the estimation results for 15 EU countries were obtained.

A common feature of the models for women and men is reflected in the positive impact of higher productivity. The main differences identified between women and men on the labor market include a different role of the selected sectors in reducing unemployment of young women and men. In the case of men, employment contributed to the reduction of unemployment rate in the construction (*F*) section. In the case of women, manufacturing (*C*) and transportation and storage (*H*) were the sections with a negative sign suggesting the youth unemployment rate reduction—along with higher share of employment in this sector. When analyzing the quality of the estimates in the model for women, the stability of the coefficient estimates in section *H* raises doubts. Therefore, this result should not be treated as the final one.

Additionally, part-time employment was observed to influence the unemployment rate in the case of women (in all specifications), however, not men. For the situation of men in individual specifications, self-employment, the level of education, or market saturation with people from the *HRST* group turned out to be important, which can be identified with the level of knowledge and technology of advancement in the national economy.

## Discussion

The presented article follows the research mainstream of econometric analyses focused on the assessment of correlations between youth unemployment rate and market and macroeconomic determinants, including economic growth and productivity of the economy, its structure in terms of NACE Rev. 2 sections as well as labor market tools. This type of research comes across certain limitations. Issue worth paying attention to is the aspect of knowledge generalization ingrained in these analyses. The strength of the presented analysis is highlighting the factors responsible for the differences in the situation of youth between and within countries. In individual countries, the situation may slightly differ from the presented estimates.

When comparing the studies available in the source literature, many of the available econometric analyzes covering the discussed problem were conducted in the first decade of the twenty-first century ((Perugini and Signorelli 2010), (Caroleo and Pastore 2007)). The presented findings cover the period after the 2008 economic crisis.

The studies differ in terms of the analysis level (country, region) and the level of data detail. Nevertheless, some conclusions can be considered as common. Similarly to the study by (Perugini and Signorelli 2010), also in this analysis the statistically significant role of part-time and self-employment contracts was demonstrated. However, in the addressed period (2008–2018) covering 28 EU countries, part-time contacts show a statistically significant impact on reducing the unemployment rate for women alone, while in the case of temporary contracts both studies did not confirm the significance.

When analyzing the results of this study, it is worth referring to the role of unemployment rate reduction assigned to individual sectors of the economy. Before the role of sectors for youth employment is discussed, it is worth emphasizing that the results of this analysis may be affected by the fact that the study covers very young people (15–24). It determines, to some extent, the type of undertaken employment. It can be their first job, sometimes occasional, which may be reflected in the employment structure. As the discussion presented by (Perugini and Signorelli 2010) shows, it may be expected that regional structural sectors biased toward low-skilled/low compensation sectors are more inclusive of young unexperienced (Pastore and Caroleo 2008) and not highly skilled workers (Quintini and Martin 2006). In turn, other authors point to the importance of economies development based on knowledge and technology sectors (Bal-Domańska and Sobczak 2020) emphasizing positive impacts of knowledge-based economy development and strengthening the quality of human capital for improving the labor market situation of young people. In this study, introducing the HRST variable to models confirmed the importance of including young people in the labor market for the development of knowledge and technology-based economies. This does not rule out the need to create jobs for people presenting different levels of qualifications. It can be stated that the development of knowledge-based economies strengthens the overall development potential of the economies by favoring the creation of jobs in various sectors, presenting diverse profiles requiring different levels of qualifications. This research confirms the importance of the general economic condition for the improvement of the situation on the labor market, also in terms of young people. The economies which include young people are the dynamically developing ones. It should also be noted that among jobseekers there are individuals with high qualifications, although, as indicated in the introduction, their transition into the labor market is more efficient.

A certain symptom of the developing economy is manifested by the growing role of employment in the construction sector, which employs many young men and, at the same time, constitutes a kind of the economic situation barometer. The crucial role of construction sector for male employment was confirmed in this study.

Taking care of the appropriate qualifications of young people and opening attractive jobs in the knowledge-based economy helps to improve the professional situation of young people. An example of the sector using new technologies to a large extent is section *J*: information and communication. This section includes the production and distribution of information and cultural products, the provision of the means to transmit or distribute these products, as well as data or communications, information technology activities and the processing of data and other information service activities. The main components of this section are publishing activities, including software publishing, radio and TV broadcasting and programming activities, telecommunications activities, information technology activities and other information service activities. This sector constitutes, by itself, a more favorable place for young people to take up professional activity. However, taking into account the results of the presented analysis and its role in explaining mainly the differences in the variance between panel units (countries), it could be noted that it links to the general condition of the economy resulting from the involvement of knowledge and technology capital.

In recent years, EU policies have placed particular emphasis on reducing the number of young people who leave school early and whose lack of experience and qualifications could make it difficult for them to take up professional activity. The analysis of the data on early leavers shows that the period 2008–2018 brought about an improvement in this respect and the number of early leavers showed a declining tendency. At the same time, a small percentage of people in this group referred to those who did not take up employment (approx. 6%); however, among them more than half of the respondents (in the EU-28 scale) expressed interest in professional activity. Reducing early school leavers and improving the education of young people in crisis situations may not be enough to reduce unemployment, but may shorten the transition period to the labor market. In this area, weaknesses are evident in the situation of Italy and Spain, which have one of the highest rates of preschool leavers and very high youth unemployment rates. In the case of Italy, it was particularly true for women.

Summarizing the research findings for young people, it is possible to formulate several recommendations for the EU policy. The first one concerns including, in the policy supporting youth employment, their high sensitivity to economic growth by supporting pro-development and anti-cyclical policies. This postulate is also formulated by other researchers, including (Bluedorn et al. 2019), (Dunsch 2016).

Another postulate refers to strengthening the policy of job creation for young workers in the sectors based on new technologies and industry. The development of industry and new technologies is conducive to economic growth and, consequently, facilitates opening new work places in service sectors, thus supporting or supplementing industrial activity.

The third postulate addresses the continuation of the policy for promoting the education of young people to provide them with the qualifications of high demand on the labor market which guarantee employment.

The final postulate concerns verifying the role of flexible forms of work in relation to higher employment of the youngest labor market participants. The analysis showed correlations between part-time contracts and the unemployment rate. Temporary contracts, in turn, do not play any significant role in improving the situation of young people; however, they affect the quality and stability of their employment. At least the flexicurity as an integrated strategy for enhancing, at the same time, flexibility and security in the labor market should be developed.

## Conclusion

The key conclusions were formulated based on answering the research questions presented at the beginning of the study.

1. The situation of youth on the labor market is closely related to the general situation in the economy and on the labor market. Growing unemployment rate can be best explained by the deterioration of the overall situation on the labor market. The increase in the adult unemployment rate translates into more than twice higher unemployment among young people. Economic growth and productivity are manifested in the reduction of unemployment rate among young people.
2. As a rule, higher employment in sections C and F is reflected by the significant reduction in the number of unemployed in the EU-28 in the years 2008–2018. In terms of the economy structure, the development of manufacturing section importance turned out to be a major factor in youth unemployment rate reduction, including women.
3. Part-time contracts have proved to be an important factor in the situation of youth on the European labor markets (EU-28). Their importance did not translate into lower unemployment rate; it was of a concurrent nature. At the same time, the economies which recorded a higher level of self-employment among young people in the analyzed period were characterized by a decline in unemployment rate.
4. As a rule, the formulated models (multiple regression) allowed explaining approximately half of the variance within panel units (countries) and only to a very small extent related to the variance between panel units (countries).

The changing economic situation observed in 2020 has contributed to the increase in unemployment rates. For the EU-27 (excluding the UK), in September 2020 against September 2019, the total unemployment rate increased by 0.9% points (up to the level of 7.5%). In the case of young people, the increase was 2.1% points (up to the level of 17.1%). In the context of the conducted analysis, the open question is how quickly the sectors most affected by the pandemic (*I*: accommodation and food service activities) will be capable of rebuilding the lost potential and whether young people will find a way to join the labor market in other sectors.

## Data Availability

The study uses the data available in the EUROSTAT database.

## Appendix

See Table

**Table 4:** List of variables (indicators)

Indicator	Description	Dimension	Source in Eurostat data base
YUN	Youth unemployment rate aged 15–24 (%)	*T*, *M*, *F*	[lfst_r_lfu3rt]
AUN	Unemployment rate of adults aged 25 and more (%)	*T*	[LFST_R_LFU3RT]
GDP	Gross domestic product at current market prices; purchasing power standard (PPS) per inhabitant	*T*	[nama_10r_3gdp]
PROD	Gross domestic product at market prices current prices; million purchasing power standards per employment (resident population concept—LFS)	*T*	[nama_10r_3gdp]
HRST	Persons employed in science and technology, from 15 to 74 years, percentage of active population	*T*	[hrst_st_ncat]
ALMP	Labor market policy expenditure by type of action; percentage of GDP (categories 2–7) (%)	*T*	[LMP_EXPSUMM]
PART	Part-time employment as percentage of the total employment for young people; aged 15–24 (%)	*T*, *M*, *F*	[yth_empl_060]
TEMP	Temporary contracts—annual data aged 15–24; percentage of total employment	*T*, *M*, *F*	[lfsi_pt_a]
TER	Young people completed tertiary education level (levels 5–8) aged 15–24 (%)	*T*, *M*, *F*	[yth_demo_040]
ELne	Early leavers from education and training—persons not employed persons aged 18 to 24 (%)	*T*, *M*, *F*	[edat_lfse_14]
SELF	Self-employment aged 15–24 (%)	*T*, *M*, *F*	[lfsa_egaps]
*L*	Participation rate in non-formal education and training (last 4 weeks) youth aged 15- 24 (%)	*T*, *M*, *F*	[yth_educ_060]
*C*	Share of employed in MANUFACTURING (C—NACE Rev. 2) aged 15–24 in total employment in this age group	*T*, *M*, *F*	[LFSA_EGAN2]
*F*	Share of employed in CONSTRUCTION (F- NACE Rev. 2) aged 15–24 in total employment in this age group	*T*, *M*, *F*	[LFSA_EGAN2]
*G*	Share of employed in Wholesale and retail trade; repair of motor vehicles and motorcycles (G—NACE Rev. 2) aged 15–24 in total employment in this age group	*T*, *M*, *F*	[LFSA_EGAN2]
*H*	Share of employed in Transportation and storage (H- NACE Rev. 2) aged 15–24 in total employment in this age group	*T*, *M*, *F*	[LFSA_EGAN2]
*I*	Share of employed in Accommodation and food service activities (I—NACE Rev. 2) aged 15–24 in total employment in this age group	*T*, *M*, *F*	[LFSA_EGAN2]
*J*	Share of employed in Information and communication (J- NACE Rev. 2) aged 15–24 in total employment in this age group	*T*, *M*, *F*	[LFSA_EGAN2]
*M*	Share of employed in Professional, scientific and technical activities (M—NACE Rev. 2) aged 15–24 in total employment in this age group	*T*, *M*, *F*	[LFSA_EGAN2]
*N*	Share of employed in Administrative and support service activities (N- NACE Rev. 2) aged 15–24 in total employment in this age group	*T*, *M*, *F*	[LFSA_EGAN2]
*O*	Share of employed in Public administration and defense; compulsory social security (O—NACE Rev. 2) aged 15–24 in total employment in this age group	*T*, *M*, *F*	[LFSA_EGAN2]
*P*	Share of employed in Education (P—NACE Rev. 2) aged 15–24 in total employment in this age group	*T*, *M*, *F*	[LFSA_EGAN2]
*Q*	Share of employed in Human health and social work activities (Q—NACE Rev. 2) aged 15–24 in total employment in this age group	*T*, *M*, *F*	[LFSA_EGAN2]
*R*	Share of employed in Arts, entertainment and recreation (R- NACE Rev. 2) aged 15–24 in total employment in this age group	*T*, *M*, *F*	[LFSA_EGAN2]
*S*	Share of employed in Other service activities (S—NACE Rev. 2) aged 15–24 in total employment in this age group	*T*, *M*, *F*	[LFSA_EGAN2]

4.

## Footnotes

1 Unfortunately, the data referring to women are only available for a limited number of countries. For 7 countries, the absence of data on self-employment for women refers to each year.

2 But not in Czechia, Croatia, Hungary, Austria, Poland, Portugal, Slovenia, where the employment rate of young people increased.

3 But not in Finland and Germany, where the employment rate of young people increased.

## References

[CR1] Bal-Domańska B (2020). The situation of youth on the european labour markets—Econometric analyses. Acta Universitatis Lodziensis Folia Oeconomica.

[CR2] Bal-Domańska, B., & Sobczak, E. (2018) Educational potential and the situation of the youth on the labour market in the European Union regions. *Double-Blind Peer-Reviewed Proceedings Part I.of the International Scientific Conference Hradec Economic Days*, 8(1): 20–31.

[CR3] Bal-Domańska B, Sobczak E, Soliman KS (2020). Econometric assessment of the relation between the situation of youth on the labour market and the macroeconomic factors among the Eu countries. Vision 2025: education excellence and management of innovations through sustainable economic competitive advantage.

[CR4] Bartosik K (2020). Temporary contracts and Okun’s law in Poland. Equilib Q J Econ Econ Policy.

[CR5] Bell A, Fairbrother M, Jones K (2019). Fixed and random effects models: making an informed choice. Qual Quant.

[CR6] Berlingieri F, Bonin H, Sprietsma M (2014) Youth unemployment in Europe-Appraisal and policy options. Centre for European Economic Research Zentrum für Europäische Zentrum für Europäische Wirtschaftsforschung GmbH (ZEW).

[CR7] Bluedorn J, Ahn J, Muhaj D, Neidlinger P, Koczan Z, Ciminelli G, An Z (2019) Improving Youth Labor Market Outcomes in Emerging Market and Developing Economies. IMF STAFF DISCUSSION NOTE, 19(02):30. https://www.imf.org/en/Publications/Staff-Discussion-Notes/Issues/2019/01/18/Work-In-Progress-Improving-Youth-Labor-Market-Outcomes-in-Emerging-Market-and-Developing-45130

[CR8] Bod’a M, Považanová M (2020). Output-unemployment asymmetry in Okun coefficients for OECD countries. J Pre-Proof Econ Anal Policy.

[CR9] Butkus M, Seputiene J (2019). The output gap and youth unemployment: an analysis based on Okun’s law. Economies.

[CR10] Calmfors L, Skedinger P (1995). Does active labour-market policy increase employment? Theoretical considerations and some empirical evidence from Sweden. Oxf Rev Econ Policy.

[CR11] Caroleo FE, Pastore F (2007) The Youth Experience Gap: Explaining Differences across EU Countries. Quaderni Del Dipartimento Di Economia, Finanza e Statistica, 41(Dezembro)/2007, Università di Perugia, Dipartimento Economia

[CR12] Clark AE, Lepinteur A (2019). The causes and consequences of early-adult unemployment: evidence from cohort data. J Econ Behav Organ.

[CR13] Dunsch S (2016). Okun’s Law and youth unemployment in Germany and Poland. Int J Manag Econ.

[CR14] Dunsch S (2017). Age- and gender-specific unemployment and Okun’s law in CEE countries. East Eur Econ.

[CR15] Eurofound. (2012). NEETs young people not in employment, education or training: characteristics, costs and policy responses in Europe. Publ Off Eur Union.

[CR16] European Commission (2011) *Youth Opportunities Initiative. * European Commission Issue COM(2011) 933 final

[CR17] Greene WH (2000) *Econometric Analysis 5th Ed.* Pearson Education International. Upper Saddle River, New Jersey, pp 07458

[CR18] Hausman JA (1978). Specification tests in econometrics. Econometrica.

[CR19] Hutengs O, Stadtmann G (2013). Age effects in Okun’s law within the Eurozone. Appl Econ Lett.

[CR20] Hutengs O, Stadtmann G (2013). Don’t trust anybody over 30: youth unemployment and Okun’s law in CEE countries. Bank i Kredyt.

[CR21] ILO (2017) Global Employment Trends for Youth 2017: Paths to a better working future. International Labour Office. Geneva (Issue October). https://www.ilo.org/global/publications/books/WCMS_737648/lang--en/index.htm

[CR22] Kliber P (2021). The diversity of Okun’s coefficient in the regions of Poland. Studia Regionalne i Lokalne.

[CR23] Korber M (2019). Does vocational education give a labour market advantage over the whole career? A comparison of the United Kingdom and Switzerland. Social Inclusion.

[CR24] Lindemann K, Gangl M (2019). The intergenerational effects of unemployment: how parental unemployment affects educational transitions in Germany. Res Soc Stratif Mobil.

[CR25] Marelli E, Choudhry MT, Signorelli M (2013). Youth and total unemployment rate : the impact of policies and institutions. Rivista Internazionale Di Scienze Sociali.

[CR26] Ngai L, Petrongolo B (2017). Gender gaps and the rise of the service economy. Am Econ J Macroecon.

[CR27] OECD, & The European Commission. (2019). *The Missing Entrepreneurs 2019*. OECD and The European Commission. 10.1787/9789264188167-en

[CR28] Parisi ML, Demidova O, Marelli E (2014) Labour Productivity of Young and Adult Temporary Workers and Youth unemployment: a Cross-country Analysis. Discussion Papers 1_2015, CRISEI, University of Naples "Parthenope", Italy. 1_2015, CRISEI, University of Naples "Parthenope", Italy

[CR29] Parisi ML, Marell E, Demidova O (2018) Youth unemployment, labour productivity and the evolution of the labour markets in Europe In: Caroleo FE, Demidova O, Marelli E, Signorelli M (eds) Young People and the Labour Market: A Comparative Perspective, 2018, Routledge, Abingdon, Oxon, UK. 10.4324/9781315178424

[CR30] Perugini C, Signorelli M (2010). Youth labour market performance in European regions. Econ Chang Restruct.

[CR31] Pompei F, Selezneva E (2019). Unemployment and education mismatch in the EU before and after the financial crisis. J Policy Model.

[CR32] Rogozińska-Pawełczyk A et al (2014) Pokolenia na rynku pracy (A. Rogozińska-Pawełczyk (ed). Wydawnictwo Uniwersytetu Łódzkiego

[CR33] Ryczkowski M, Zinecker M (2020) Gender unemployment in the Czech and Polish labour market. Argumenta Oeconomica 2:213–229. 10.15611/aoe.2020.2.09

[CR34] Sahnoun M, Abdennadher C (2019). Labor market institutions and performance economic within trial labor market models: flexibility, rigidity, and flexicurity. Rev Black Polit Econ.

[CR35] van Ark B, Frankema E, Duteweerd H (2004) Productivity and employment growth: an empirical review of long and medium run evidence. In GGDC Research Memorandum (Background Working Paper for the World Employment Report 2004 International Labour Office, Geneva, Issue No. GD-7)

[CR36] Votinius JJ (2014). Young employees: securities, risk distribution and fundamental social rights. Eur Labour Law Journal.

[CR37] Whitehouse G (1992). Legislation and labour market gender inequality: an analysis of OECD countries. Work Employ Soc.

[CR38] Williams DR (2004). Youth self employment: Its nature and consequences. Small Bus Econ.

[CR39] Wooldridge JM (2010) Econometric Analysis of Cross Section and Panel Data, The MIT Press

